# Application of Quantitative Magnetic Resonance Imaging in the Diagnosis of Autism in Children

**DOI:** 10.3389/fmed.2022.818404

**Published:** 2022-05-12

**Authors:** Shilong Tang, Lisha Nie, Xianfan Liu, Zhuo Chen, Yu Zhou, Zhengxia Pan, Ling He

**Affiliations:** ^1^Department of Radiology, Children’s Hospital of Chongqing Medical University, National Clinical Research Center for Child Health and Disorders, Ministry of Education Key Laboratory of Child Development and Disorders, Chongqing Key Laboratory of Pediatrics, Chongqing, China; ^2^GE Healthcare, MR Research China, Beijing, China

**Keywords:** children, autism, iron, magnetic resonance imaging, brain

## Abstract

**Objective:**

To explore the application of quantitative magnetic resonance imaging in the diagnosis of autism in children.

**Methods:**

Sixty autistic children aged 2–3 years and 60 age- and sex-matched healthy children participated in the study. All the children were scanned using head MRI conventional sequences, 3D-T1, diffusion kurtosis imaging (DKI), enhanced T2*- weighted magnetic resonance angiography (ESWAN) and 3D-pseudo continuous Arterial Spin-Labeled (3D-pcASL) sequences. The quantitative susceptibility mapping (QSM), cerebral blood flow (CBF), and brain microstructure of each brain area were compared between the groups, and correlations were analyzed.

**Results:**

The iron content and cerebral blood flow in the frontal lobe, temporal lobe, hippocampus, caudate nucleus, substantia nigra, and red nucleus of the study group were lower than those in the corresponding brain areas of the control group (*P* < 0.05). The mean kurtosis (MK), radial kurtosis (RK), and axial kurtosis (AK) values of the frontal lobe, temporal lobe, putamen, hippocampus, caudate nucleus, substantia nigra, and red nucleus in the study group were lower than those of the corresponding brain areas in the control group (*P* < 0.05). The mean diffusivity (MD) and fractional anisotropy of kurtosis (FAK) values of the frontal lobe, temporal lobe and hippocampus in the control group were lower than those in the corresponding brain areas in the study group (*P* < 0.05). The values of CBF, QSM, and DKI in frontal lobe, temporal lobe and hippocampus could distinguish ASD children (AUC > 0.5, *P* < 0.05), among which multimodal technology (QSM, CBF, DKI) had the highest AUC (0.917) and DKI had the lowest AUC (0.642).

**Conclusion:**

Quantitative magnetic resonance imaging (including QSM, 3D-pcASL, and DKI) can detect abnormalities in the iron content, cerebral blood flow and brain microstructure in young autistic children, multimodal technology (QSM, CBF, DKI) could be considered as the first choice of imaging diagnostic technology.

**Clinical Trial Registration:**

[http://www.chictr.org.cn/searchprojen.aspx], identifier [ChiCTR2000029699].

## Background

Autism spectrum disorder (ASD) is a group of neurodevelopmental disorder with the onset in early childhood, characterized by impairment in communication, and social interaction, rigidity of interests, and repetitive stereotypical behaviors (1–4). The incidence of ASD is increasing worldwide, while the etiology and pathogenesis of autism are poorly understood and no drug is available (5, 6). Children with autism are mainly treated with rehabilitation intervention, and there is no specific treatment yet. Studies have shown that early and reasonable systematic rehabilitation intervention can improve the symptoms of most children, and even obtain a basic “recovery.” The earlier the rehabilitation intervention, the better the recovery of children, which requires clinicians to accurately diagnose children with autism at an early stage (6, 7).

Currently, the early diagnosis of ASD is mainly based on the patients’ clinical manifestations, but the clinical manifestations of most ASD children are atypical and the symptoms-based early diagnosis is difficult, especially for low-age children with ASD, which eventually leads to many ASD children missing effective early intervention expectations. Therefore, there is an urgent need for early, fast, convenient and feasible diagnostic methods for ASD (8–11).

In previous studies, only a few researchers explored whether the brain blood flow or gray matter of ASD children was abnormal by 3D-pseudo continuous Arterial Spin-Labeled (3D-pcASL), quantitative susceptibility mapping (QSM) and diffusion kurtosis imaging (DKI) sequences (7, 12–15). For example, Tang et al. (13) found a decrease in iron content in some brain regions of ASD children. Mori et al. (14) confirmed that the cerebral blood flow in some brain areas of ASD children decreased. McKenna et al. (15) demonstrated that gray matter microstructure in some brain regions of autistic children was abnormal. However, they did not prove whether there is spatial overlap in brain regions with reduced iron content, cerebral blood flow perfusion, abnormal gray matter microstructure, and whether there is a correlation between them.

In this study, we performed multimodal MRI (conventional MRI sequences, ESWAN, DKI, 3D-pcASL, and 3D-T1 MRI sequences) to scan the brains of autistic children and healthy children. The quantitative maps of the QSM, DKI, and CBF MRI data and a 3D-T1 structure map of the brain were obtained through post-processing. The quantitative and structural maps of the two groups were compared and analyzed. The correlation was analyzed to obtain a specific MRI diagnostic basis of autism in children if any differences were found.

## Materials and Methods

### Ethics Statement

The study protocol was approved by the Human Ethics Committee of the Children’s Hospital of Chongqing Medical University (No. 2018-47). Written informed consent was obtained from the parents or guardians of all the patients before the examinations.

### Patients

From June 2018 to December 2020, we recruited 65 2- to 3-year-old autistic children at the Children’s Hospital of Chongqing Medical University as the research group. All the children were tested for iron trace elements, hemoglobin, and the red blood cell volume at our hospital. The inclusion criteria of the study group were as follows: body mass index between 15 and 18; right handedness; fulfillment of the diagnostic criteria of autism in the American Diagnostic and Statistical Manual of Mental Disorders (DSM-IV); no evidence of neurological functional diseases; no evidence of other organ-associated diseases; no evidence of other diseases that may affect brain function and structure; no history of drug treatment in the past; and no abnormality in routine MRI examinations of the head. Five children snored with excessive breathing activity after sedation, resulting in image quality defects. Finally, the data of 60 children (32 male and 28 female; mean age, 2.72 years; mean body mass index, 16.89) were included in the study.

During the same period, we recruited 63 healthy children of matched age and sex as the control group, and all the children were tested for iron trace elements, hemoglobin, and the red blood cell volume at the laboratory in our hospital. The inclusion criteria of the control group were as follows: body mass index between 15 and 18; right handedness; no evidence of neurological functional diseases; no evidence of other organ-associated diseases; no evidence of other diseases that may affect brain function and structure; no abnormality in routine MRI examination of the head; iron trace elements in venous blood within the normal range (6.5–9.85 μmol/L); and a hemoglobin value and mean corpuscular volume (MCV) value in the normal range (Hb, 110–150 g/L; MCV, 80–100 fl). Three children snored and breathed with excessive motion after sedation, resulting in image quality defects. Finally, 60 children (31 male and 29 female; average age, 2.76 years; average body mass index, 16.54) were included in the study.

### Imaging Data Collection

Data collection was performed in the MRI room of Children’s Hospital of Chongqing Medical University. A GE Discover MR750 3.0T MR scanner (GE Medical Systems, Milwaukee, WI, United States) and an 8-channel head and neck coil were used to scan the head with routine MRI sequences, 3D-pcASL, DKI, ESWAN, and 3D-T1 sequences. All the children were examined after sedation and sleeping in Sedation Center (sedation mode: Dexmedetomidine nasal drip, 2 ug/kg). The routine sequence included sagittal T2, transverse T2, T1 Flair, T2 Flair, and DWI; FOV, 24 cm, matrix of acquired images, 256*256, slice thickness, 5 mm, and 16 layers, total scanning time, 4 min and 32 s. 3D-pcASL: FOV, 24 cm; slice thickness, 4 mm; TR, 4628 ms; PLD, 1525 ms; slab, 1; and 36 layers; scanning time, 4 min and 29 s. DKI: FOV, 24 cm; matrix of acquired images, 128*128, slice thickness, 3 mm; TR, 4,500 ms; 43 layers; total diffusion direction, 30; and B values of 0, 1,000, 2,000; scanning time, 5 min and 20 s. ESWAN: FOV, 24 cm; slice thickness, 3 mm; TR, 81.8; flip angle, 20; TE, 4 ms; Locs per slab, 50; slab, 1; scanning time, 7 min and 47 s. 3D-T1: FOV, 24 cm; slice thickness, 1 mm; TR, 7.9 ms; flip angle, 12; TE, 3.1 ms; and 156 layers; scanning time, 3 min and 43 s. Before the children were scanned, the conventional sequence and study sequence were scanned once using a special magnetic resonance head water model (Model 2152220; MRS Sphere with Solution; GE, Milwaukee, WI, United States). When the image signals were uniform, the children were examined again.

### Imaging Data Post-processing

**FIGURE 1:**
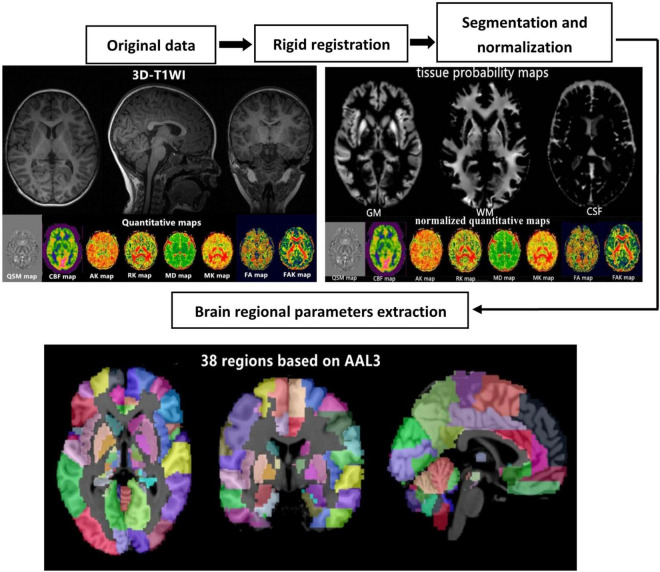
Schematic diagram of the image processing and parameter extraction.

To calculate the quantitative parameters, including the gray matter volume (GMV), white matter volume (WMV), QSM, cerebral blood flow (CBF), mean diffusivity (MD), fractional anisotropy (FA), fractional anisotropy of kurtosis (FAK), radial kurtosis (RK), mean kurtosis (MK), and axial kurtosis (AK) values of different brain regions, we followed an atlas-based image processing approach (Figure 1). Quantitative susceptibility mapping calculation was conducted with the phase ESWAN images using a publicly available implementation tool (STI Suite version 3.0).^1^ The CBF, MD, FA, FAK, MK, RK, and AK maps were further calculated from the 3D-pcASL and DKI data using an AW4.6 GE Workstation. Second, we performed a rigid registration between the QSM/ASL/DKI quantitative maps and 3D-T1WIs using SPM12 software^2^ based on MATLAB (MathWorks, Natick, MA, United States). Third, 3D-T1WIs were segmented and non-linearly normalized into the MNI space using the CAT12 toolbox^3^ implemented in SPM12 to obtain the tissue probability maps and normalized QSM/ASL/DKI quantitative maps. Finally, the brain was parcellated into 38 anatomical regions based on automated anatomical labeling atlas (AAL-3) for quantitative parameter extraction. The brain regional volumes, QSM, CBF, and DKI, including MD, FA, FAK, MK, AK, and RK, can be extracted by averaging the values from voxels in specific brain regions. The estimated global GMV and WMV were further normalized by correction of the intracranial volume.

### Statistical Analysis

SPSS 25.0 statistical software was used, and the measurement data are expressed as mean ± standard deviation. All data were tested for normality and variance homogeneity prior to analyses, and the independent two-sample *T*-test was used if the data conform to the normality and variance homogeneity. The brain volume, iron content and cerebral blood flow data in the same brain area of children in the study group and control group were compared by *T*-test for two independent samples. The DKI data, the AK, MD, FA, FAK, MK, and RK values, in the same brain region of children in the study and control groups were compared by *T*-test for two independent samples. The iron, hemoglobin and red blood cell volume data of children in the study and control groups were compared by *T*-test for two independent samples.

Receiver operating characteristic (ROC) curve analysis was performed to evaluate the diagnostic value of ASD children. The area under the curve (AUC) of more than 0.5 indicated significant diagnostic value, and the AUC value closer to 1 was indicative of the better diagnostic value.

## Results

### Comparison of the Cerebral Blood Flow and Iron Content Measurement Data Between the Study and Control Groups

**FIGURE 2:**
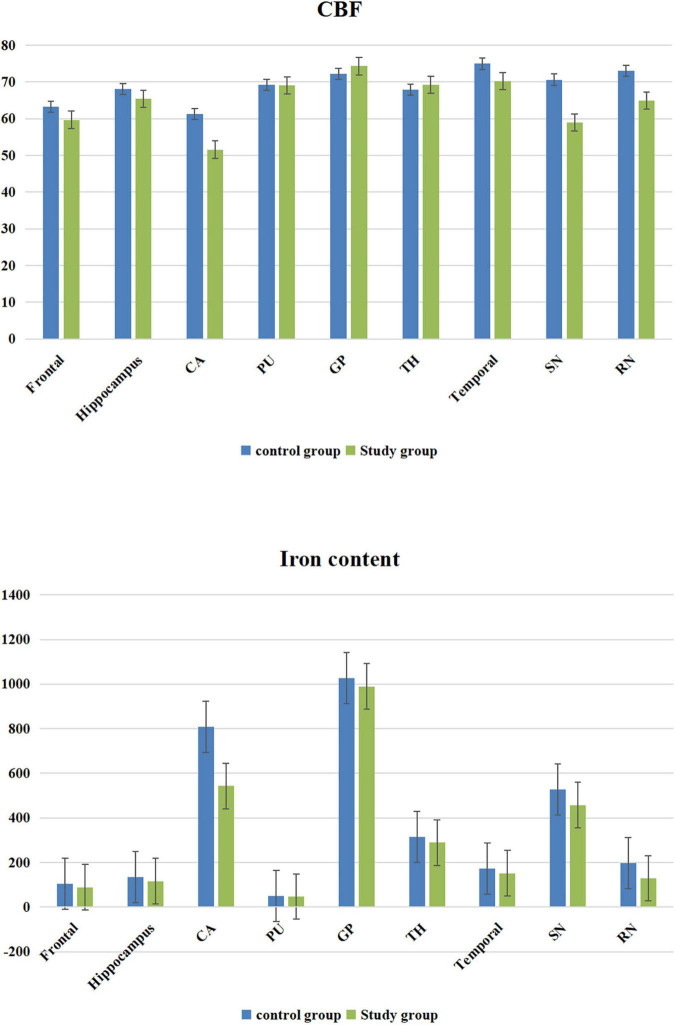
Cerebral blood flow (CBF) value and iron content bar graphs.

The CBF value and iron content of the frontal lobe, temporal lobe, hippocampus, caudate nucleus, substantia nigra and red nucleus in the study group was significantly lower than that of the corresponding brain areas in the control group (*P* < 0.05) (Table 1 and Figure 2).

**TABLE 1:** Comparison of the cerebral blood flow (CBF) and iron content measurement data between the study and control groups [$\bar{x}$± s, *n* = 60].

		Frontal	Hippocampus	CA	PU	GP	TH	Temporal	SN	RN
CBF (ml/100 g⋅min)	Control group	63.2 ± 8.63	68.07 ± 13.38	61.20 ± 16.51	69.26 ± 21.99	72.20 ± 37.18	67.88 ± 17.28	74.95 ± 15.61	70.64 ± 27.98	72.99 ± 30.20
	Study group	59.69 ± 9.91	65.43 ± 12.19	51.57 ± 9.01	69.10 ± 13.43	74.30 ± 20.24	69.18 ± 13.07	70.29 ± 11.45	59.01 ± 18.28	64.95 ± 22.19
	*T*-value	8.623	1.993	5.783	7.287	3.991	4.564	11.875	2.973	6.442
	*P*-value	0.001	0.004	0.003	0.358	0.297	0.189	0.001	0.002	0.001
Iron content ppb(× 10^–14^)	Control group	104.94 ± 15.59	135.77 ± 15.48	807.57 ± 35.79	49.51 ± 13.87	1025.64 ± 163.78	314.64 ± 19.65	172.99 ± 18.47	528.62 ± 48.52	197.63 ± 29.66
	Study group	89.32 ± 15.94	116.44 ± 16.17	543.24 ± 37.63	47.59 ± 15.66	989.74 ± 143.25	289.57 ± 20.62	151.82 ± 20.4	457.45 ± 39.57	129.58 ± 31.87
	T value	1.687	3.896	7.692	0.985	5.639	1.003	2.438	1.392	6.781
	*P*-value	0.001	0.001	0.002	0.067	0.135	0.094	0.001	0.001	0.001
										

### Comparison of the Diffusion Kurtosis Imaging Results Between the Study and Control Groups

**FIGURE 3:**
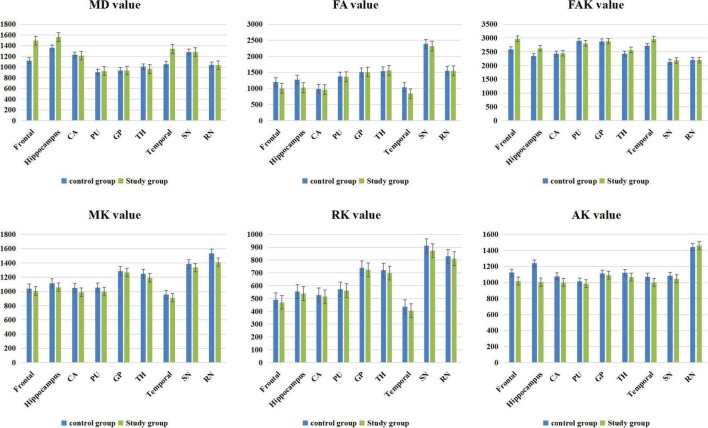
Diffusion kurtosis imaging (DKI) value bar graphs.

The MK value of the frontal lobe, temporal lobe, putamen, hippocampus, caudate nucleus, substantia nigra, and red nucleus of the study group was significantly lower than that of the corresponding brain areas in the control group (*P* < 0.05). The RK and FA values of the frontal lobe, temporal lobe, and hippocampus in the study group were significantly lower than those in the corresponding brain regions in the control group (*P* < 0.05). The MD and FAK values of the frontal lobe, temporal lobe, and hippocampus in the control group were significantly lower than those in the corresponding brain regions in the study group (*P* < 0.05). The AK values of the frontal lobe, temporal lobe, hippocampus and caudate nucleus in the study group were significantly lower than those in the corresponding brain regions of the control group (*P* < 0.05) (Table 2 and Figure 3).

**TABLE 2:** Comparison of the diffusion kurtosis imaging results between the study and control groups [10^–4^ μm^2^/ms$,\bar{x}$± s, *n* = 60].

		Frontal	Hippocampus	CA	PU	GP	TH	Temporal	SN	RN
MD value	Control group	1121.89 ± 50.88	1361.07 ± 92.92	1229.51 ± 141.55	908.63 ± 115.87	937.33 ± 117.72	1007.63 ± 129.89	1053.64 ± 42.74	1282.43 ± 116.17	1042.22 ± 186.25
	Study group	1494.05 ± 61.18	1561.49 ± 93.85	1211.10 ± 138.95	926.68 ± 95.15	936.37 ± 135.43	964.42 ± 118.48	1339.92 ± 42.15	1283.30 ± 126.94	1036.29 ± 259.37
	T value	−5.687	−4.325	12.568	−6.899	15.689	7.661	−8.334	−5.667	4.235
	*P*-value	0.001	0.008	0.912	0.681	0.561	0.112	0.001	0.763	0.881
FA value	Control group	1198.67 ± 182.35	1276.54 ± 163.33	987.56 ± 129.39	1373.87 ± 166.03	1504.81 ± 268.13	1538.58 ± 223.78	1040.43 ± 163.72	2390.88 ± 246.12	1550.65 ± 178.43
	Study group	1009.27 ± 168.54	1025.36 ± 188.23	965.92 ± 149.36	1362.33 ± 148.07	1513.62 ± 278.33	1563.21 ± 225.12	838.54 ± 196.24	2317.37 ± 221.56	1547.28 ± 163.54
	T value	9.874	7.679	4.056	7.213	−5.623	−7.934	5.213	6.653	7.327
	*P*-value	0.001	0.001	0.704	0.569	0.726	0.829	0.001	0.127	0.128
FAK value	Control group	2584.56 ± 238.57	2340.52 ± 198.25	2430.70 ± 153.47	2896.67 ± 301.84	2874.59 ± 269.13	2429.83 ± 298.65	2714.91 ± 263.68	2131.81 ± 273.63	2205.98 ± 314.67
	Study group	2969.32 ± 235.31	2621.87 ± 221.55	2443.76 ± 178.62	2803.59 ± 288.62	2884.59 ± 258.33	2563.87 ± 271.19	2958.98 ± 312.54	2187.56 ± 269.64	2197.65 ± 268.61
	T value	−12.673	−3.897	−8.971	7.467	−12.634	−15.768	−3.876	−6.629	9.762
	*P*-value	0.001	0.001	0.973	0.089	0.691	0.098	0.001	0.691	0.988
MK value	Control group	1041.27 ± 247.96	1113.77 ± 186.48	1048.89 ± 211.49	1055.84 ± 166.45	1285.79 ± 255.22	1247.02 ± 256.28	955.04 ± 154.82	1385.06 ± 229.23	1531.84 ± 232.72
	Study group	1008.08 ± 244.34	1060.71 ± 201.83	989.46 ± 226.40	997.80 ± 187.32	1268.37 ± 241.20	1192.24 ± 245.56	912.12 ± 158.52	1333.43 ± 224.45	1410.35 ± 214.26
	T value	15.689	11.238	7.652	9.994	1.893	5.557	11.294	22.567	3.894
	*P*-value	0.003	0.006	0.001	0.001	0.786	0.235	0.004	0.001	0.001
RK value	Control group	490.93 ± 125.81	554.48 ± 125.28	527.55 ± 129.82	573.87 ± 125.03	738.87 ± 168.25	721.56 ± 133.73	435.97 ± 143.70	910.46 ± 129.35	829.94 ± 125.61
	Study group	471.25 ± 165.92	538.90 ± 131.40	515.36 ± 149.13	562.33 ± 153.07	724.42 ± 178.73	699.33 ± 125.66	406.57 ± 163.45	873.33 ± 121.22	811.49 ± 149.55
	T value	8.376	2.229	3.051	4.223	3.684	1.996	11.237	9.671	2.364
	*P*-value	0.001	0.000	0.356	0.079	0.751	0.088	0.001	0.114	0.856
AK value	Control group	1123.69 ± 92.44	1239.29 ± 107.22	1077.73 ± 105.12	1014.02 ± 168.27	1112.05 ± 171.21	1121.25 ± 159.94	1074.11 ± 99.98	1084.05 ± 139.48	1443.03 ± 196.92
	Study group	1017.30 ± 87.74	1004.66 ± 75.26	1001.43 ± 115.05	986.46 ± 178.21	1093.91 ± 168.64	1069.11 ± 156.75	1002.44 ± 101.99	1047.12 ± 145.24	1461.41 ± 197.39
	T value	5.662	2.897	4.336	6.879	2.337	11.562	7.821	1.982	4.287
	*P*-value	0.001	0.001	0.000	0.568	0.236	0.983	0.001	0.561	0.894
										

### Comparison of the Brain Volume Measurement Data Between the Study and Control Groups

The volume of the caudate nucleus in the study group was significantly smaller than that in the control group (*P* < 0.05). No significant difference was found in the volume of whole brain between the study and control groups (*P* > 0.05) (Table 3).

**TABLE 3:** Comparison of the brain volume measurement data between the study and control groups [cm^3^, $\bar{x}$± s, *n* = 60].

	Whole brain	Frontal	Hippocampus	CA	PU	GP	TH	Temporal	SN	RN
Control group	1356.72 ± 92.38	143.69 ± 15.73	9.15 ± 0.62	8.51 ± 0.86	14.15 ± 1.17	2.41 ± 0.35	13.21 ± 1.06	126.25 ± 8.02	0.77 ± 0.12	0.52 ± 0.09
Study group	1329.20 ± 114.26	143.16 ± 12.25	9.29 ± 0.59	8.26 ± 0.76	14.22 ± 1.19	2.39 ± 0.33	13.29 ± 1.12	125.50 ± 7.70	0.78 ± 0.11	0.52 ± 0.07
T value	1.894	7.832	3.652	9.763	2.883	3.765	6.892	9.764	5.732	9.421
*P*-value	0.256	0.136	0.057	0.002	0.109	0.372	0.903	0.078	0.062	0.147
										

### Comparison of the Test Results of the Trace Iron, Hemoglobin Content, and Red Blood Cell Volume Between the Study and Control Groups

No significant difference was found in the trace iron, hemoglobin content and red blood cell volume between the study and control groups (*P* > 0.05) (Table 4).

**TABLE 4:** Comparison of the test results of the trace iron, hemoglobin content, and red blood cell volume between the study and control groups [$\bar{x}$± s, *n* = 60].

	Trace iron (μmol/L)	Hemoglobin (g/L)	MCV (fL)
Control group	7.89 ± 0.61	139.42 ± 25.58	83.62 ± 5.16
Study group	7.92 ± 0.57	141.73 ± 24.57	86.45 ± 4.89
T value	12.376	13.871	4.579
*P*-value	0.382	0.971	0.089

### Correlation Analysis

**FIGURE 4:**
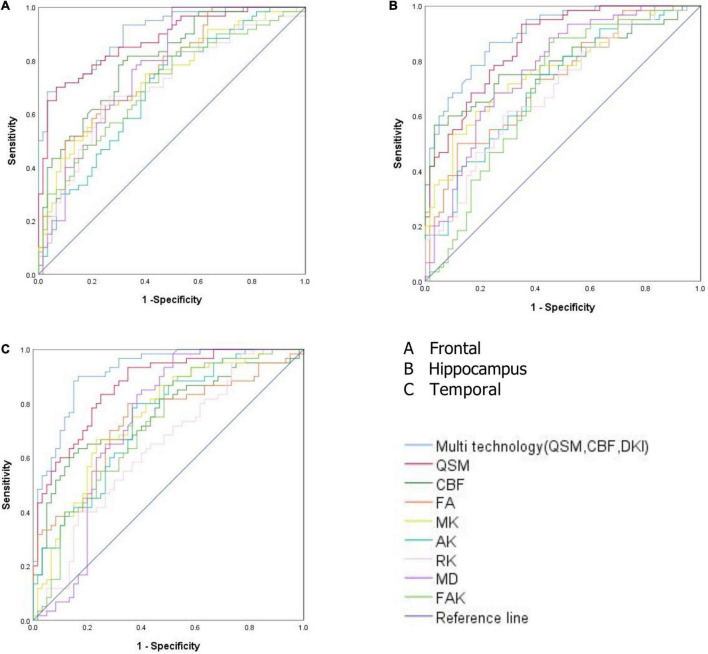
**(A)** Receiver operating characteristic (ROC) curve analysis results of quantitative susceptibility mapping (QSM), cerebral blood flow (CBF), diffusion kurtosis imaging (DKI), and multimodal technology (QSM, CBF, DKI) values in frontal lobe. **(B)** ROC curve analysis results of QSM, CBF, DKI, and multimodal technology (QSM, CBF, DKI) values in hippocampus. **(C)** ROC curve analysis results of QSM, CBF, DKI, and multimodal technology (QSM, CBF, DKI) values in temporal lobe.

The values of CBF, QSM, and DKI in frontal lobe, temporal lobe and hippocampus could distinguish ASD children (AUC > 0.5, *P* < 0.05), among which multimodal technology (QSM, CBF, DKI) had the highest AUC (0.917) and DKI had the lowest AUC (0.642) (Table 5 and Figure 4).

**TABLE 5:** Receiver operating characteristic (ROC) curve analysis results of quantitative susceptibility mapping (QSM), cerebral blood flow (CBF), and diffusion kurtosis imaging (DKI) values in frontal lobe, temporal lobe and hippocampus (*n* = 60).

Variable	AUC	Std. error	*P*-value	95% CI	
				
				Lower bound	Upper bound
**Frontal**					
Multimodal technology (QSM, CBF, DKI)	0.902	0.026	0.000	0.850	0.953
QSM	0.876	0.031	0.000	0.815	0.937
CBF	0.803	0.039	0.000	0.726	0.880
FA	0.760	0.043	0.000	0.676	0.844
MK	0.737	0.045	0.000	0.649	0.826
AK	0.691	0.048	0.000	0.597	0.785
RK	0.711	0.047	0.000	0.618	0.804
MD	0.771	0.043	0.000	0.686	0.855
FAK	0.709	0.047	0.000	0.616	0.801
**Hippocampus**					
Multimodal technology (QSM, CBF, DKI)	0.896	0.027	0.000	0.842	0.950
QSM	0.860	0.032	0.000	0.797	0.924
CBF	0.789	0.042	0.000	0.706	0.872
FA	0.729	0.045	0.000	0.641	0.818
MK	0.763	0.043	0.000	0.678	0.848
AK	0.708	0.047	0.000	0.616	0.800
RK	0.690	0.048	0.000	0.597	0.784
MD	0.769	0.043	0.000	0.685	0.854
FAK	0.692	0.049	0.000	0.595	0.788
**Temporal**					
Multimodal technology (QSM, CBF, DKI)	0.917	0.025	0.000	0.868	0.965
QSM	0.868	0.032	0.000	0.806	0.930
CBF	0.760	0.044	0.000	0.673	0.847
FA	0.733	0.046	0.000	0.642	0.823
MK	0.752	0.044	0.000	0.666	0.839
AK	0.738	0.045	0.000	0.650	0.826
RK	0.642	0.05	0.007	0.543	0.741
MD	0.729	0.049	0.000	0.632	0.825
FAK	0.716	0.047	0.000	0.624	0.808

## Discussion

Most of the routine brain MRI results of children with autism are negative, which is not helpful for the early diagnosis of children with autism (16, 17). In this study, we performed multimodal MRI scans on the brains of children with autism, and obtained the microstructures of the brains through image post-processing to understand whether the brain microstructure of children with autism is abnormal and obtain the criteria for early objective imaging diagnosis of the brain of children with autism.

Quantitative susceptibility mapping (QSM) is a relatively new technology developed based on SWI imaging technology in recent years. Compared with traditional SWI based on magnetic susceptibility differences, QSM can detect tiny changes in the iron content in the brain and provide better specific spatial image contrast; thus, it has higher sensitivity and credibility (18–22). 3D-pcASL technology is an MR perfusion imaging technology, it uses water molecules in the blood in the artery as endogenous contrast agents for vascular perfusion imaging. The images of blood water molecules reaching the target at the threshold level for imaging before and after being labeled are collected, and the two images are subtracted to obtain the final image with blood perfusion information (23–29). The iron content and cerebral blood flow in the frontal lobe, temporal lobe, hippocampus, caudate nucleus, substantia nigra, and red nucleus in the autistic children was lower than that in the healthy children. The reason for the differences may be the decrease in the iron content in these brain regions due to insufficient cerebral blood flow in the autistic children (30, 31). In the human body, iron exists mainly in the form of hemoglobin iron and non-hemoglobin iron. Hemoglobin iron exists mainly in the form of oxygenated hemoglobin, deoxyhemoglobin, and methemoglobin in the blood, accounting for approximately 2/3 of the total iron content in the human body. The levels of myoglobin and various enzymes are very small. Non-hemoglobin iron includes ferritin and hemosiderin in blood. More than 1/3 of non-pigment iron in the brain exists in the form of ferritin. The decrease in cerebral blood flow in the brain regions described above leads to a decrease in hemoglobin iron and non-hemoglobin iron in the blood of these regions, leading to a decrease in the iron content in these above brain regions (30–32).

Diffusion kurtosis imaging (DKI) is a magnetic resonance imaging technique that reflects the non-Gaussian distribution of water molecules in brain tissue. It introduces kurtosis (K) defined in terms of probability and statistics and is used to solve the problem of fiber direction at fiber intersections, which cannot be solved by the second-order tensor, and to quantify the degree of water molecular diffusion deviating from a Gaussian distribution (33–37). The comparison of MK values of DKI parameters showed that the MK value of the frontal lobe, temporal lobe, putamen, hippocampus, caudate nucleus, substantia nigra, and red nucleus in the autistic children was lower than that of the corresponding brain regions in the healthy children. The MK value depends on the structural complexity of the tissues in the region of interest. More complex structures exhibit more significant the limitations of the diffusion of non-normally distributed water molecules and larger MK values. Therefore, the low MK value in some brain regions of the autistic children may be caused by the tight and irregular brain tissue structure and destruction of tissue structure complexity, indicating that microstructural changes may have occurred in these brain regions. The comparison of DKI parameters showed that the MD and FAK values of the frontal lobe, temporal lobe and hippocampus in autistic children were higher than those in the control group, while the FA value was lower than that in the control group. The increase in the MD value and decrease in the FA value in the brains of autistic children may indicate damaged axonal integrity due to an increase in axonal membrane permeability, axonal microstructure damage, or neurogenesis defects. The increase in the FAK value may be due to neuron remodeling and the loss of special fiber bundles, changes in fiber composition or an increase in cell permeability, which all can lead to a change in the FAK value.

The AK and RK values of the frontal lobe, temporal lobe and hippocampus in the autistic children were lower than those in the healthy children. AK and RK are the average values of diffusion kurtosis parallel and perpendicular to the long axis of the diffusion tensor, respectively. Both measures describe information related to the direction of the DKI and enable comprehensive observations of micropathological changes in biological tissue structures. AK and RK are sensitive to changes in gray matter microstructure. The decrease in AK and RK values may be due to the destruction of myelin integrity, nerve fiber density and parallelism and disorder of a microstructure. In the study, we selected only the six aforementioned parameters for the DKI study because the MD, FAK and FA values represent white matter structure parameters and MK, AK, and RK values represent gray matter parameters. The results showed that all six parameters in the frontal lobe, temporal lobe, hippocampus, and other brain regions were changed, indicating that microstructures in both gray matter and white matter in the brain regions of interest in the autistic children were changed.

The blood test results showed no significant difference in the trace iron, hemoglobin content or red blood cell volume between the autistic children and healthy children (*P* > 0.05). However, QSM showed that the iron content in some brain regions of the autistic children was decreased (*P* < 0.05). This finding indicates that the iron content in some brain regions may have begun to decline with no obvious change in blood trace element iron detected in the early stage of autism in these children and that QSM technology is more sensitive to changes in the iron content in the brain regions of autistic children. Additionally, the volume of the caudate nucleus in the autistic children was lower than that in the healthy children (*P* < 0.05), but no significant difference was found in other brain regions (*P* > 0.05). However, a decrease in cerebral blood flow occurred in all the brain areas of the autistic children with a decreased iron content. This finding suggests that the decrease in the iron content in some brain regions of the autistic children may not be related to the volume in the brain regions but to the decrease in blood flow in these brain regions. The results show that the frontal lobe, temporal lobe and hippocampus not only have a decreased iron content and decreased cerebral blood flow but also have changes in the brain microstructure. Therefore, the frontal lobe, temporal lobe and hippocampal regions in autistic children may be the first to undergo changes in brain microstructure. These three brain regions may be the key areas in the brain images that can be used to diagnose autism in children.

The frontal lobe, temporal lobe, hippocampus and other brain regions in the autistic children not only showed a decreased iron content and decreased CBF but also a change in brain microstructure. We speculate that the frontal lobe, temporal lobe, and hippocampal regions may be the first areas to show microstructural changes in autistic children. The values of CBF, QSM, and DKI in frontal lobe, temporal lobe and hippocampus could distinguish ASD children (AUC > 0.5, *P* < 0.05), among which multimodal technology (QSM, CBF, DKI) had the highest AUC (0.917) and DKI had the lowest AUC (0.642). Therefore, these three brain regions could be served as the key areas of brain imaging in the diagnosis of ASD children, and multimodal technology (QSM, CBF, DKI) could be considered as the first choice of imaging diagnostic technology.

The frontal lobe is the most developed lobe of the brain, it determines the language ability and thinking ability of a person, if the development of the frontal lobe is damaged, these functions will be affected (38, 39). The main function of the temporal lobe is related to language comprehension, memory, and mental activity, if a person suffers from temporal lobe injury, he can’t understand the meaning of words spoken by other people and himself, and is unable to understand other people’s questions (40, 41). The hippocampus plays a role in memory and spatial orientation, and damage to the hippocampus can lead to memory decline and loss of sense of direction and perception (42). The results of the study show that the iron content and cerebral blood flow in the frontal lobe, temporal lobe, hippocampus, and other brain regions of autistic children are lower than those of healthy children, and the DKI value is also abnormal. The abnormal microstructure of the above brain regions may lead to poor development of these three brain regions in autistic children, eventually causing poor development of language, thinking, motor, comprehension, and other aspects in autistic children.

In the past, other researchers have found that the brain regions of autistic children that often undergo microstructural changes are mostly in the frontal lobe, temporal lobe, hippocampus, basal ganglia, and the above brain regions are closely related to children’s language development and intellectual development. Therefore, this study selected the above brain regions for prior study, and whether there are microstructural changes in other brain regions will be studied in the next step.

### Limitations of This Study

First, we found that the autistic children who were diagnosed earlier and were given targeted treatment showed a higher attenuation rate. Therefore, we selected only 2- to 4-year-old children; other age groups were not included in the study and need to be studied in future research. Second, we collected only the data of autistic children in the local region. Thus, we did not perform a multicenter study; therefore, our data were limited to a certain extent.

In conclusion, the iron content and cerebral blood flow in the frontal lobe, temporal lobe, and hippocampus of autistic children are lower than those of healthy children, and the brain microstructures in the children with autism are changed. Quantitative magnetic resonance imaging (including QSM, 3D-pcASL, and DKI) can detect abnormalities in the iron content, cerebral blood flow and brain microstructure in young autistic children, multimodal technology (QSM, CBF, DKI) could be considered as the first choice of imaging diagnostic technology.

## Data Availability Statement

Publicly available datasets were analyzed in this study. This data can be found here: https://figshare.com/s/4250d86ec77711b8c50a.

## Ethics Statement

The studies involving human participants were reviewed and approved by Institutional Review Board of Children’s Hospital of Chongqing Medical University. Written informed consent to participate in this study was provided by the participants’ legal guardian/next of kin.

## Author Contributions

ST: experimental design, project management, and statistics. LN: software support. XL: statistical analysis and image analysis. ZC and YZ: data acquisition and data analysis. ZP and LH: experimental design and project management. All authors contributed to the article and approved the submitted version.

## Conflict of Interest

LN was employed by GE Healthcare. The remaining authors declare that the research was conducted in the absence of any commercial or financial relationships that could be construed as a potential conflict of interest.

## Publisher’s Note

All claims expressed in this article are solely those of the authors and do not necessarily represent those of their affiliated organizations, or those of the publisher, the editors and the reviewers. Any product that may be evaluated in this article, or claim that may be made by its manufacturer, is not guaranteed or endorsed by the publisher.

## Footnotes

1
https://people.eecs.berkeley.edu/∼chunlei.liu/software.html

2
http://www.fil.ion.ucl.ac.uk/spm/

3
http://www.neuro.uni-jena.de/cat/

## Abbreviations

MRI: magnetic resonance imaging
DKI: diffusion kurtosis imaging
3D-pcASL: 3D-pseudo continuous arterial spin-labeled
ASD: autism spectrum disorder
QSM: quantitative susceptibility mapping
DSM-IV: diagnostic and statistical manual of mental disordersfourth edition
MCV: mean corpuscular volume
VBM: voxel-based morphology
CBF: cerebral blood flow
ESWAN: enhanced T2*- weighted magnetic resonance angiography
MK: mean kurtosis
RK: radial kurtosis
AK: axial kurtosis
MD: mean diffusivity
FAK: fractional anisotropy of kurtosis
FA: fractional anisotropy.
